# Hinging Prejudices and Stereotypes in Mathematics

**DOI:** 10.1007/s11245-025-10194-4

**Published:** 2025-04-26

**Authors:** Jordi Fairhurst, José Antonio Pérez-Escobar

**Affiliations:** 1 Departamento de Filosofía y Trabajo Social, Universitat de les Illes Balears, Palma, Spain; 2 KU Leuven, Leuven, Belgium; 3 Department of Philosophy, University of Geneva, Geneva, Switzerland; 4 Department of Logic, History and Philosophy of Science, UNED, Madrid, Spain

**Keywords:** Mathematical hinges, Mathematical practices, Hinge epistemology, Testimonial injustice, Prejudices

## Abstract

This paper develops a theoretical framework to better understand how implicit biases about social identity (e.g., gender, race, class, seniority, or institutional affiliation) may influence different stages of knowledge production. To do so, it makes use of hinge epistemology to describe how inter- (results of applications of mathematical rules) and extra-mathematical (e.g., stereotypes and prejudices) factors play a role in our mathematical practices and knowledge production. Accordingly, we will describe how these different factors confer or remove normative power from mathematical pieces in a broad economy of credibility. By doing so, we intend to unify two strands of hinge epistemology that have hitherto been separate: that of mathematical practices and that of testimonial justification. The upshot of this proposal is the development of a theoretical framework that enables more effective, appropriately informed measures to ameliorate both epistemic injustice in social contexts and epistemic harm within mathematics.

## Introduction

Knowledge production within the domain of mathematics requires practitioners to implicitly presuppose a wide array of rules that guarantee the correct functioning of their epistemic operations (such as justifying, proving, doubting, or criticizing). For example, mathematicians often need to take certain axioms and standards of proof for granted to produce and evaluate (potential) knowledge.

Since mathematics is often portrayed as a value-free discipline, there may be a temptation to conclude that all presuppositions regulating mathematics must share this neutrality. However, recent research has called this conclusion into question, emphasizing how social aspects and power dynamics implicitly regulate mathematical practices (Ernest 2021; Pérez-Escobar and Sarikaya 2022; Hunsicker and Rittberg 2022). Implicit biases about social identity can (positively or negatively) influence knowledge production within mathematics by, say, determining whose work is of mathematical interest, what papers should be published in journals, or who should be selected for academic events and positions.

Any attempt to minimize the interference of these implicit biases within knowledge production processes in mathematics first requires a good theoretical story about what they are and how they function. More specifically, we need to better understand what epistemic features are exhibited by implicit biases and what normative force they exert over mathematicians and their practices.

This paper aims to address this lacuna by developing a theoretical framework that describes how implicit biases about social identity (e.g., gender, race, class, seniority, or institutional affiliation) influence different stages of mathematical knowledge production. For the purposes of this paper, we will focus on the processes of certification and petrification in mathematics. Accordingly, we set out to provide a rational mechanism that spells out how mathematics is petrified and certified by the influence of both intra (e.g., results of the application of mathematical rules) and extra-mathematical (e.g., stereotypes and prejudices) factors. The upshot of this proposal is the development of a theoretical groundwork that enables more effective, appropriately informed measures to ameliorate both epistemic injustice in social contexts and epistemic harm within mathematics.

To do so, the paper makes use of hinge epistemology to describe how positive and negative identity-stereotypes interfere in our mathematical practices. The reasons for choosing hinge epistemology are twofold. First, it provides powerful theoretical tools to make sense of the fundamental presuppositions implicitly exerting normative force over our epistemic practices, making it possible for us to perform epistemic operations and regulating how we behave and judge. Thus, it is an enticing theoretical framework to make sense of implicit biases within mathematics. Second, the power of these theoretical tools has been successfully demonstrated with regards to both mathematics (McGinn 1989; Moyal-Sharrock 2004; Kusch 2016; Martin 2022; Fairhurst et al. 2024) and implicit biases (especially, prejudices) in testimonial exchanges separately (Coliva 2015; Boncompagni 2019; 2024). However, the overlap between these domains remains unexplored. Thus, our proposal is an enticing venture, as it aims to fill a knowledge gap within the existing literature on hinge epistemology, while shedding light on how implicit bias can shape mathematical practices.

The structure of the paper is thus. Initially, it provides a brief overview of hinge epistemology (Sect. 1) and its application to mathematics (Sect. 2). Subsequently, it motivates the need for a theoretical account of the prejudices and stereotypes that implicitly regulate mathematical practices (Sect. 3). Accordingly, a hinge-account of negative identity-stereotypes, i.e., prejudices, (Sect. 3.1) and positive identity-stereotypes (Sect. 3.2) is developed. Lastly, we exemplify the applications of this theoretical framework with a toy-case. Specifically, (i) we explain how prejudicial and stereotypical hinges can influence epistemic practices at different stages of mathematical knowledge production and (ii) we spell out what epistemic outcomes result from this influence (Sects. 4 and 5).

## Hinge Epistemology

The basis for our proposal is a theoretical framework known as ‘hinge epistemology’. Hinge epistemology is an umbrella-term for a diverse group of epistemic theories that integrate Wittgenstein’s idea of hinges in *On Certainty* (Wittgenstein 1969) in the development of a viable epistemology.

The central claim shared by these theories is that epistemic practices do not take place in a vacuum, but rather turn on commitments, propositions or rules functioning like hinges. Just as the hinges must stay put for a door to move, so too must these commitments, propositions or rules be exempt from doubt for our epistemic practices to work. They are the frame of reference that enables our epistemic practices to function. Doubting hinges would be cognitively disastrous: stifling inquiry, preventing the attainment of epistemic goods, or leading us to systematically doubt many propositions. Hinges, then, can be broadly understood as:

The **fundamental presuppositions** of a domain of inquiry;

That are exempt from doubt;

And make it **possible** for us to perform **epistemic operations** (e.g., judging, discovering, justifying, verifying, or evaluating beliefs as rational).

A clear-cut example of hinges are rules of evidential significance (cf. Coliva 2015). Suppose that within an area of inquiry, an epistemic agent sets up a cognitive project: a question-procedure pair to attain epistemic goods (cf. Wright 2014). For instance, they ask ‘Is there a computer screen in front of me?’ and stipulate a procedure (e.g., using their visual perception) to attain an epistemic good (e.g., a true justified belief concerning how the world is). The success of this project is (partly) dependent on there being hinges guaranteeing the proper functioning of the epistemic operations at stake.^1^

Among other things, the agent must accept a rule of evidential significance stipulating the chosen procedure as a legitimate way of finding an answer to the question. For example, the epistemic agent would need to accept that their sense of visual perception provides them with reliable information about the world, allowing them to form true justified beliefs from what they observe. Here, ‘Sense perception is reliable’ functions as a hinge. It is:

A **fundamental presupposition** of an area of inquiry, implicitly assumed in kindred cognitive projects. Moreover, it is assumed without justification, as there is no cognitive project providing evidence for it.^2^

**Beyond doubt,** as doubt would be cognitively disastrous: stifling inquiry, preventing the attainment of epistemic goods, and leading to systematic doubt about all visual perceptions.

**Enabling our epistemic operations** by normatively governing what constitutes evidence for belief and stipulating what procedures are reliable in knowledge production. For example, the epistemic agent can acquire and assess the required evidence to justify the empirical claim ‘There is a computer in front of me’.

The above toy-case exemplifies how hinge epistemology provides enticing theoretical tools to bring out the fundamental presuppositions implicitly and normatively regulating epistemic practices. It emphasizes how hinges exert normative force and control over our judgements and/or behaviors in said context. For example, ‘Sense perception is reliable’ regulates how the epistemic agent undertakes cognitive projects for inquiry, acquires and assesses evidence to respond to a question, the procedures they implement to attain epistemic goods, the way they go about justifying their empirical claims, and so on. Such tools have been successfully implemented in the analysis of the implicit presuppositions regulating epistemic practices in numerous domains, such as science, religion, ethics, gender, or politics (e.g., Wright 2014; Herman 2015; O’Hara 2018; Boncompagni 2024; Erisken et al. 2022; Sandis and Moyal-Sharrock 2022).

## Mathematical Hinges

Among the applications of hinge epistemology, we find the domain of mathematics. In *On Certainty*, Wittgenstein himself alludes to a wide variety of examples, some of them mathematical, to elucidate the notion of hinge. Although scholars disagree over the precise features (e.g., epistemic or non-epistemic, justified or unjustified) and scope (e.g., whether hinges are axioms, other mathematical rules and propositions, or it depends on the context) of mathematical hinges (see McGinn 1989; Moyal-Sharrock 2004; Kusch 2016; Martin 2022; Fairhurst et al. 2024), there is widespread consensus over the existence of mathematical hinges regulating mathematical practices.

For instance, Wittgenstein discusses Moore’s example of ‘there is a hand’ to showcase how casting doubt on some propositions in certain contexts is harmful because it prevents the attainment of (sometimes basic and essential) epistemic goods. He exemplifies how in different manners, but we will adapt this case to make it relevant for mathematics. Let us say we aim to articulate a cognitive project for the practice of counting fingers, which in turn can be used to learn basic arithmetic, as children typically do.

**Question**: How many fingers does my fully extended hand display?

**Procedures**: Counting.

**Epistemic good**: Learning facts about my fingers, learning arithmetic.

What needs to be taken for granted for our epistemic procedure to be a legitimate way of answering the question and attaining the desired epistemic goods? This, of course, depends on the context, as we will argue later on. But in most contexts, ‘there is a hand’ cannot be subjected to the skeptic’s doubt, since this would prevent the attainment of epistemic goods. Eventually, knowledge and epistemic practices rest on this premise, and some of these premises, more distinctively mathematical, may play a similar role: ‘my hand has 5 fingers’ (or my counting may become erratic when I subtract 2 fingers) or ‘5 - 2 = 3’. Although what constitutes a mathematical hinge is contested in the literature, a typical intuition is that axioms play this precise role: they are not to be doubted and contested, but they allow the attainment of further mathematics that builds on them. An example is the Euclidean axiom that ‘the whole is greater than the part’: this is exempted from doubt and just accepted to do some mathematics, like proofs in Euclidean geometry. However it also provides an essential intuition in mundane practices, like in the case of counting fingers: we must accept that the hand or the five fingers of the hand (the whole) is greater than individual fingers for our finger arithmetic, and more broadly, rules of addition and subtraction, to work as desired.^3^ However, as explained above, mathematics that build on paradigmatic hinges (not just axioms) may acquire similar roles. In any case, as we see, hinges are not falsifiable but regulate knowledge production and (in ideal cases) make it possible and catalyze the attainment of epistemic goods.

Moreover, the theoretical framework of hinge epistemology allows us to describe many of the nuances of mathematical practices and education. For example, very recently, it has been suggested that the character of mathematical propositions (whether they are hinges of some type or non-hinges) is dynamic and context-sensitive, and that these contextual changes in the character of mathematical propositions can steer mathematics in one direction or another (Fairhurst et al. 2024). This is in line with the later Wittgenstein’s tenet that the meaning of a proposition resides in its use, and its use varies across contexts. This study has also suggested that the ‘right’ status of the different mathematical propositions involved in a given practice accounts for the success of mathematics at different stages, like education and research. For instance, mathematics education would be inefficient if the presentation of formal mathematics at university relied mostly on non-hinges or ordinary propositions instead of propositions immunized from doubt, i.e., hinges. This is the case especially because such mathematical propositions often violate intuitions typically acquired before university. For instance, for many years, young students hold the intuition ‘the shortest distance between two points is a straight line’ in mundane contexts and when doing Euclidean geometry. However, in their first non-Euclidian geometry course they need to let go of both the parallel postulate and their mundane intuitions about distance. These regulated their previous activities just like ‘there is a hand’ in the case of counting fingers. In this new context, they are presented with alternatives and need to accept them as the new ground (i.e., hinges of new practices). They must, however, resort to the previous grounds (i.e., hinges) in the previous practices. Failing to do this would be akin to questioning whether there is a hand when learning to count fingers.

From this, it follows that a distortion of the epistemic characteristics of sets of mathematical propositions can interfere with the success of mathematical practices. Furthermore, because there is no ultimate ground to decide the epistemic role of a given proposition in a given context but this is instead open-ended, such distortions may happen without much realization and extra-mathematical factors—like social factors (see Sect. 3)—may be involved Overall, these contextual dynamics further put mathematics in line with other epistemic practices, even ordinary practices. For instance, ‘there is a hand’ can be an ordinary proposition or a hinge depending on the context; if we were to count our fingers, doubting whether there is a hand would impair this counting practice.

To better illustrate the role of hinges in mathematics and their open-endedness, consider the case presented by Fairhurst et al. (2024; see Sects. 4 and 5). Basically, Euler’s conjecture for polyhedra (namely, that the number of vertices minus the number of edges plus the number of vertices equals 2) constituted a regularity based on counting polyhedra time and time again. According to Wittgenstein's later philosophy of mathematics, regularities like this ‘petrify’ and, thus, acquire normative power. At its most basic level, this petrification consists of repeated experiences with empirical phenomena involving arithmetic and related practices like counting, so that empirical regularities ‘petrify’ into mathematical propositions. After the process, instead of being falsifiable by experience, the propositions become normatively charged and regulate experience. A very simple and paradigmatic example is putting two apples and two apples together and the eventual petrification of 2 + 2 = 4: if we counted only 3, we would not falsify the mathematical proposition but use it to regulate our counting (e.g., by looking for the missing apple). Note the resemblance to the counting of edges, vertices and faces in polyhedra. Petrification, however, also applies to areas of mathematics with no clear empirical components, because repeated experience with elements of pure mathematics like calculations, proofs, models and intuitions can also immunize them against doubt (Steiner 2000; Pérez-Escobar et al. 2024).^4^ Back to the case of Euler’s conjecture: its normative power immunized it from falsification after counterexamples were presented: instead of falsifying the conjecture, it regulated what counts as a polyhedron and what does not. This happened, at least in part, due to Euler’s credentials as a mathematician.^5^ This case was famously discussed by Lakatos in *Proof and Refutations* (Lakatos 1976). In this work we follow up on this lead and make the case that the contextual dynamics of mathematical hinges rely, at least in part, on issues concerning testimony and the credibility of knowledge producers.

## A Hinge-Account of Prejudices and Stereotypes

Mathematics has often been portrayed as a value-free discipline: the epistemic ideal upon which we ought to model all scientific endeavors. On this account, one may be tempted to conclude that any hinge regulating our epistemic practices within mathematics must share this neutrality. However, recent research suggests that mathematics cannot be divorced from sociological aspects implicitly exerting normative force over its epistemic practices (see Ernest 2021; Pérez-Escobar and Sarikaya 2022; Hunsicker and Rittberg 2022 for an overview). Consider the case of social identities (e.g., gender, race, class, seniority, or institutional affiliation) and their related power dynamics.

These sociological aspects not only play an important role in academic formation (e.g., who is able to access undergraduate studies in mathematics or complete the required graduate studies to become a researcher) but also influence different stages of knowledge production. For example, social identity and power dynamics can contribute to who can attain membership in mathematical communities conducting research, what mathematical ideas are chosen as relevant and worthy of pursuit or how the quality of mathematical ideas is assessed. ‘Works on the explanatoriness of proofs (D’Alessandro 2019), the purity of methods (Detlefsen and Arana 2011), on mathematical beauty (Thomas 2016), publishability of results (Geist et al. 2010) all show this’ (Hunsicker and Rittberg 2022, p. 51, 164).

This paper aims to develop a theoretical framework to better understand how implicit biases about social identity (e.g., gender, race, class, seniority, or institutional affiliation) may influence different stages of knowledge production. Initially, it outlines Boncompagni’s (2019; 2024) hinge-account of identity-prejudices (Sect. 3.1). Subsequently, it extends this hinge-account to another social identity bias: positive identity-stereotypes (Sect. 3.2). Lastly, we exemplify the applications of this theoretical framework with a toy-case where we explain (i) how prejudicial and stereotypical hinges can influence epistemic practices at different stages of mathematical knowledge production and (ii) what epistemic outcomes result from this influence (Sects. 4 and 5).

### Prejudicial-Hinges

Seminal works by Collins (2000), Dotson (2011; 2014), Fricker (2007), Hooks (1992), Medina (2013), Moraga and Anzaldúa (1981), Pohlhaus (2012; 2017) and Spivak (2003), among others, have inspired a generation of philosophers to study how prejudice impacts our epistemic exchanges and harms epistemic agents in both their capacity to learn and contribute to knowledge. Consider the case of testimonial exchanges.

Testimony is an indispensable source of knowledge production and dissemination. Much of what we know about the world (e.g., its history, what it contains, what happens in it) is acquired through testimony from others. A central feature within testimonial exchanges is the *economy of credibility*: the distributions in who we choose to trust or not (see Fricker 2007, 30–58 for an overview). There are numerous factors informing our judgments about the credibility of an epistemic agent. Some are fair (e.g., track record of reliability, expertise) and should be rationally considered (see Sect. 3.2). Others are unfair and should be rationally excluded (see Sect. 3.1). Clear-cut examples are *identity-prejudices*: widely held disparaging associations between a social group and one or more negative attributes.

Because of holding identity-prejudices about a social group, we unjustly undervalue the trustworthiness of those epistemic agents who are members of the social type in question. This results in an *epistemic injustice*: the injustice one suffers when being diminished as an epistemic agent or knower. More specifically, it is a case of *testimonial injustice*, occurring when an epistemic agent receives an unfair deficit of credibility because of identity-prejudices (related to, say, race, gender, or class) in the receiver’s judgment.^6^ Testimonial injustice not only deprives its victim from fulfilling their role as an epistemic agent, it also negatively affects the perpetrator (and the broader epistemic community). By attributing an unfair deficit of credibility to an agent because of identity prejudices, the perpetrator may ignore relevant testimony and good evidence.

Although identity-prejudices are central to testimonial injustice, it is unclear what normative power these prejudices exert within the epistemic structure of testimonial justification. Boncompagni (2019; 2024) suggests that hinge epistemology provides a viable theoretical framework to spell out the normative power of identity-prejudices. To do so, initially, she provides a detailed analysis concerning how identity-prejudices exhibit features characteristic of hinges.^7^

Identity-prejudices, first, are presuppositions within a variety of (unjust) testimonial exchanges. They are background assumptions operating without doxastic mediation (and often without justification) in cognitive projects to determine the (un)trustworthiness of epistemic agents.^8^ Second, they are exempt from doubt insofar as they are resistant to counterevidence (see Allport 1954; Appiah 1990; Fricker 2007; Boncompagni 2019; 2024 for a defense). Third, they enable epistemic operations by regulating behaviors and/or judgments within the domain of testimonial exchanges. More specifically, they are norms of ‘evidential significance, determining what does and what does not count as evidence for belief’ (Boncompagni 2024, p. 294). For example, a listener holding identity-prejudices will judge the speaker as untrustworthy and their testimony as lacking evidential significance.

Drawing on the parallelism between hinges and identity-prejudices, Boncompagni demonstrates how hinge epistemology serves as a means for understanding how prejudice works epistemically. Consider an epistemic exchange between Alex—the speaker—offering a testimony about P to Dani—the hearer. Dani is confronted with the immediate task of gauging whether they are justified in believing what they have been told by Alex. Dani’s task can be reformulated in terms of a cognitive project:

**Question**: Is P true?

**Procedure**: Alex’s testimony.

**Epistemic good**: A justified belief about P.

The success of this project depends on the procedure being a legitimate way of answering the question and delivering the desired epistemic goods. In other words: Alex must be perceived as trustworthy (i.e., sincere and knowledgeable with respect to P) and their testimony must be regarded as being of evidential significance.

Our epistemic perception of an agent’s credibility in a testimonial exchange is not usually the result of deliberation, but rather we directly perceive them as trustworthy or not in the light of various background assumptions, i.e., hinges (Boncompagni 2019, pp. 174; 2024, 294; cf. Fricker 2007, pp. 36–36, 71; Coliva 2015; Forthcoming). Accordingly, what is needed is a hinge serving as a rule of evidential significance that stipulates Alex’s testimony as a reliable source of information.

In ordinary circumstances, this is accomplished through the *testimonial-hinge* ‘T is a reliable informant on this occasion’ (Coliva 2015; Forthcoming).^9^ This is:

A **basic trust presupposition** regarding the reliability of the informant on a specific occasion that is accepted without justification in unproblematic testimonial exchanges;

**Beyond doubt**, as doubt would be cognitively disastrous: stifling inquiry, preventing the attainment of epistemic goods, and leading us to systematically doubt all testimonies.

**Enabling our epistemic operations** by normatively governing what constitutes evidence for belief and stipulating testimony as a reliable procedure in knowledge-production. For example, Dani can acquire and assess the evidence provided by Alex to justifiably believe in P.

However, what happens when these ordinary circumstances are subverted? Suppose Alex is a woman of color and Dani is a white male adhering to racist and misogynist ideologies. Due to Dani’s ideologies, he holds a prejudicial-hinge that associates Alex’s social type (i.e., woman of color) with disparaging attributes (e.g., epistemically unreliable).^10^ This prejudicial-hinge is a taken-for-granted presupposition that works as a norm of evidential significance, regulating Dani’s judgments and/or behavior by (unjustly) deeming Alex as an untrustworthy epistemic agent and her testimony as lacking evidential significance. Dani’s cognitive project now lacks the testimonial-hinge granting evidential status to Alex’s testimony. Instead, he has a prejudicial-hinge deeming the epistemic procedure as illegitimate and stopping the attainment of epistemic goods. Accordingly, Dani will no longer be justified in believing P in virtue of Alex’s testimony.

So, although Dani would be justified in believing what he is told by Alex, the prejudicial-hinge prevents testimonial justification. How does the prejudicial-hinge exert this normative power within the epistemic structure of testimonial justification? Boncompagni (2024, pp. 295–301; cf. Keren 2007; Zagzabski 2012; Begby 2021) argues that this normative power is best explained in terms of *preemption*. Prejudicial-hinges do not remove testimonial justification once it has been obtained. They are not defeaters that lead to *ex post* belief revision or higher order doubt when the hearer becomes aware of certain circumstances (e.g., the speaker’s social type). Instead, prejudicial-hinges intervene before the possibility of testimonial justification arises. They preempt evidence by means of *source discrediting*, excluding *ex ante* information derived from a certain epistemic agent. Accordingly, the testimonial-hinge, which in an unprejudiced environment guarantees an epistemic agent to function as a reliable source of information, is no longer present. In its place, there is a prejudicial-hinge functioning as an evidential gatekeeper, discrediting the epistemic agent as a reliable source of (testimonial) evidence and preventing the attainment of epistemic goods (i.e., a justified belief about P in virtue of the speaker’s testimony).^11^ The preemption exerted by this prejudicial-hinges is local, applying only to those testimonial exchanges where the speaker pertains to the social group targeted by the prejudice.

### Stereotypical-Hinges

Thus far we have examined prejudicial-hinges: negative identity-stereotypes implicitly presupposed in testimonial exchanges that normatively regulate what counts as (testimonial) evidence. Prejudices, however, are not the only kind of identity-stereotypes epistemic agents can hold.

Identity-stereotypes can be broadly understood as widely held associations between a social group and one or more attributes, solely in virtue of the group’s identity (e.g., gender, race, class, sexual orientation, and so on). Although all identity-stereotypes are empirical generalizations about a given social group, they can carry positive or negative valence (or both).^12^ Throughout this section we want to consider the positive valenced counterparts of prejudicial-hinges.^13^ Specifically, we suggest that hinge epistemology provides a viable theoretical framework to spell out the normative power of positive identity-stereotypes within the epistemic structure of testimonial justification.

Consider, again, the case where Dani is confronted with the immediate task of gauging whether he is justified in believing in P solely in virtue of what he has been told by Alex. As we know, the success of Dani’s project is dependent on there being hinges guaranteeing that the epistemic operation (i.e., Alex’s testimony) is a legitimate way of answering his question and attaining epistemic goods. This time, Dani holds a positive identity-stereotype associating Alex’s social type with positive attributes (e.g., epistemic reliability). For example, suppose Alex is Canadian and Dani holds that Canadians are polite and, therefore, do not lie. Here, Dani’s positive identity-stereotype is a taken-for-granted presupposition that works as a norm of evidential significance. Specifically, it deems Alex as a trustworthy agent and their testimony as evidentially significant. Accordingly, Dani now holds the required hinge to utilize his epistemic procedure (i.e., Alex’s testimony) as a legitimate way of answering his question and attaining epistemic goods (i.e., a justified belief about P).

At first glance, it may seem that Dani simply holds the testimonial-hinge (where Alex is T):

**Testimonial-Hinge**: T is a reliable informant on this occasion.

Coliva proposes the testimonial-hinge in an attempt to capture the *minimal conditions* that must be fulfilled to have testimonial justification. This accomplished through her embrace of *Local Moderatism*, which holds that ‘in order for a subject S to be justified in believing that p, based on attester T’s saying so to her, it must be the case that, absent defeaters, the hinge that T is a reliable informant on that occasion is assumed’ (Coliva 2015, p. 64). What we obtain is a testimonial-hinge where justification is both weak (i.e., easily defeasible) and local (i.e., it applies solely to T, not other people). Nevertheless, this hinge seems intuitively present in our epistemic exchanges, capturing testimonial justifications we ordinarily take ourselves to have (Coliva 2015, p. 64).

Upon closer inspection, it is clear Dani assumes more than the testimonial-hinge allows. Specifically, Dani’s hinge is not local but tailored to Alex’s social identity. What justifies Alex’s testimony is not ‘T is trustworthy’ but ‘People like T are trustworthy’ (cf. Boncompagni 2024, p. 295). Although Coliva (2015, p. 64) concedes that we often do more than merely assume the testimonial-hinge, imposing these further and ‘more stringent conditions would deprive us of many testimonial justifications we ordinarily take ourselves to have’. Thus, we require more than Coliva’s testimonial-hinge to describe how Dani attains testimonial justification.^14^

We suggest characterizing Dani’s hinge as a *stereotypical testimonial-hinge*. On this account, testimonial justification is the result of assuming the minimal conditions of Coliva’s testimonial-hinge together with a positive identity-stereotype about the testifier’s social group. Thus, we obtain:

**Stereotypical Testimonial-Hinge (hereinafter, ST-Hinge)**: T is a reliable informant on this occasion because they pertain to the social group Y.

The normative force of the ST-Hinge is also one of *preemption*. Like prejudicial-hinges, ST-hinges do not intervene once testimonial justification has been obtained. They ought not be understood as instances where the hearer employs positive identity-stereotypes to revise or amplify the testimonial evidence obtained previously through a testimonial hinge. Instead, ST-hinges take the place of the testimonial-hinge, thereby playing a central role in qualifying the evidential status of a testimony.

However, unlike prejudicial-hinges, ST-hinges preempt by means of *source crediting*. They deem a testifier as a reliable source of information solely in virtue of their membership in a group about which the hearer holds a positive identity-stereotype. For example, Dani credits Alex as a reliable source of (testimonial) evidence because they are Canadian, thereby enabling the attainment of epistemic goods (i.e., a justified belief about P in virtue of the speaker’s testimony). The preemption exerted by Dani’s ST-hinge is local, applying only to those testimonial exchanges where the speaker pertains to the social group targeted by the positive identity-stereotype.

Having obtained a hinge account of positive identity-stereotypes and clarified how they exert normative power within the epistemic structure of testimonial justification, it is important to distinguish between two types of ST-hinges.

Positive identity-stereotypes can be either reliable or unreliable. The former refer to reliable empirical generalizations that can be rightfully and justly used when making credibility judgments. For example, that quantum physicists are good at mathematics is a positive identity-stereotype that reflects a reliable empirical generalization and, as such, can be rightfully used when making credibility judgments about these individuals when providing us with a testimony about the result of a mathematical operation. When a ST-hinge involves a reliable positive identity-stereotype, we will call it a *reliable-ST-hinge*.

**Reliable-ST-hinge***:* T is a reliable informant on this occasion because they pertain to the social group Y, and our empirical generalization about Y is reliable.

Reliable ST-hinges, then, are norms of evidential significance that result in positive epistemic outcomes, granting testimonial justification to those who rightfully deserve it.

By contrast, the latter refers to unreliable empirical generalizations that can distort our credibility judgments about a testifier by giving them a credibility excess, thereby committing epistemic wrongs or injustices (cf. Medina 2011; Davis 2016; Yap 2017). For example, that white men are trustworthy informants is an unreliable empirical generalization that can distort our credibility judgments about this social group, thereby providing them with testimonial justification in contexts where it is neither warranted nor justified. Within the group of unreliable positive identity-stereotypes we also include those stereotypes that are utilized as backhanded compliments. Namely, cases where being positively stereotyped in one domain (e.g., women are warmer than men) typically leads to being correspondingly negatively stereotyped in another domain (e.g., women are less competent than men) (see Fiske et. al. 2002). When a ST-hinge involves an unreliable positive identity-stereotype, we will call it an *unreliable-ST-hinge*:

**Unreliable-ST-hinge**: T is a reliable informant on this occasion because they pertain to the social group Y, and our empirical generalization about Y is unreliable.

Unreliable ST-hinges, then, are norms of evidential significance that result in negative epistemic outcomes, granting testimonial justification to those who do not deserve it, thereby causing epistemic wrongs or injustices.

## Dialogical Interactions and “Certification” as Another Source of the Normative Power of Mathematical Hinges

Having developed a hinge-account of testimony, prejudices and stereotypes (Sect. 3), hereinafter we exemplify the applications of our theoretical framework to the case of mathematics. To do so, initially, we introduce Dutilh-Novaes’ (2021) ‘Prover-Skeptic Model’ to better describe how testimonial evidence can play a central role in knowledge production in mathematics (cf. Geist et al. 2010; Andersen 2017). Subsequently, we extend and utilize this model to describe how prejudicial-hinges and (reliable and unreliable) ST-hinges may influence mathematicians and their practices at different stages of mathematical knowledge production.

As we hinted in the section above, there are likely extra-mathematical factors, like social identifiers, that play a role in whether mathematical propositions are used as hinges or not in a given context. A novelty of this work is the consideration of many of these factors not in isolation but in the more realistic setting where they co-exist with other extra and intra-mathematical modulators of normativity. As such, these factors individually considered are not determinant in how a mathematical proposition is used in a given situation, but may pull towards one direction in what may be called an ‘economy of credibility’.^15^ In this section we will discuss a process involving extra-mathematical factors relevant to the acquisition of normative power in mathematics and to such an overall economy of credibility: the dialogical interactions in mathematical practices.

It has been argued that dialogical interactions are essential for knowledge production, including contexts of disagreement and controversy in mathematics, where they can be highly productive (e.g. Dutilh-Novaes 2021; Ernest 2023). In the context of mathematical practices, Dutilh-Novaes (2021) uses the terms ‘prover’ and ‘skeptic’ in her ‘Prover–Skeptic model’ for each role respectively, while Ernest (2023) calls these figures ‘proponent’ and ‘critic’ (the scope of ‘proponent’ is wider than that of ‘prover’; more on this later). Following Ernest, who argues that these two figures suffice for a minimal characterization of dialogical interactions, our analysis will also focus on the ‘proponent’ and ‘critic’. Of course, other dialogical interactions are possible and may increase the complexity of the scheme that we will present in this section.

It is easy to see how cooperative interactions can be productive (e.g. a co-authored paper combines the insight and expertise of different mathematicians). However, competitive interactions can be positive too. For instance, participation barriers in the form of gatekeeping (where the ‘critics’ or ‘skeptics’ are journal and conference referees, journal editors, PhD examiners and so forth) contribute to ‘epistemological hygiene’ by casting out cronyism, external interests and corruption in general in knowledge acceptance processes (Ernest 2021, 2023). Competitive interactions can also improve knowledge in attack-and-defense cycles: in addressing the challenges posed by the critic, the proposer yields more polished knowledge. The latter somewhat blurs the line between cooperation and competition, this being a reason for which some authors talk about ‘adversarial collaboration’ in knowledge production (Mellers et al. 2001; Dutilh-Novaes 2021; Cleeremans 2022; Rakow 2022). In the case of mathematics, adversarial collaboration contributes to the ‘certification’ of proofs, a sort of trust metric that the community attributes to a given proof (Dutilh-Novaes 2021). This is because the more a proof resists checking and testing by the community, the more reliable it is perceived to be and the more unlikely it is for mathematicians to subsequently question it or the conjecture it proves.

Lakatos’ *Proof and Refutations* already hinted at this sometimes unintuitive dialectic:

Then not only do refutations act as fermenting agents for proof-analysis, but proof-analysis may act as a fermenting agent for refutations! What an unholy alliance between seeming enemies! (Lakatos 1976, 48)

However, it is reasonable that the certification processes described by Dutilh-Novaes (2021) apply to other mathematical elements other than proofs. For instance, Ernest (2023) shows the relevance of these dialogical interactions for the construction and reception of mathematical knowledge in general, even including metatheoretical reflections of mathematical knowledge. While he concedes that dialogical interactions are particularly interesting for proofs because their main purpose is persuasive, he claims that settling ‘whether the concepts or procedures in a new theory are coherently defined and defensibly valid’ (Ernest 2023, p. 5) falls under similar dynamics. The dialogical interactions to which Lakatos (1976) refers involve not just proofs, but concepts, definitions and conjectures. Another angle to look at this is that ‘open textured’ concepts and/or negotiable elements, inasmuch as their implementation in practices are outcomes of iterative dialogical processes (and can be proposed, challenged, defended and polished), are subjected to certification processes. Thus, this is one of the principles that account for mathematical practices to take one form of another despite the open-endedness of at least several parts of mathematics. There is recent literature arguing for the open texture of mathematical concepts and rules (Tanswell 2018, Zayton 2022; Fairhurst et al. 2024; Pérez-Escobar (forthcoming)), and the selection of some definitions over competing ones on practical grounds (Cellucci 2018; Coumans and Consoli 2023). Furthermore, because adversarial collaboration also takes place in the sciences, it is expectable that some sort of certification (unrelated to proof but closer to empirical validation) happens for mathematical models as well (e.g. some components of the least certified models may be less trusted, since they have not been tested and refined much), and thus the notion of certification may be extended for more applied mathematics too. Accordingly, we will adopt the term ‘proponent’ as a broader version of ‘prover’, and ‘mathematical piece’ as a broader term than ‘proof’.

Overall, we have that the (sustained) certification of mathematical pieces (not just proofs) increases their credibility in an overall economy of credibility, synergizing with the petrification of mathematical regularities in this endeavor. In some contexts, this may trigger a normative use of a mathematical piece, as a hinge that instead of being subjected to refutation or abandonment, regulates mathematical practices. Note that this synergy is a continuous process, but the outcome (namely, what kind of use is made of a mathematical piece in a given context) can be discrete or even binary.^16^ The higher the combined contribution of certification and petrification (as different processes that confer normative charge to mathematics), the more the contexts where the mathematical piece will be constitutive of a hinge. It is worth remembering here that testimony is a source of evidence, but as we see, its dynamics also regulate evidence.

It is precisely these dialogical interactions and certification processes that, as we will argue in Sect. 5, are the key to unifying the hinge epistemologies of prejudices/stereotypes and mathematics. Before we get there, we first must briefly survey the recent literature on epistemic injustice in mathematics relating to certification processes.

The most immediate concern in dialogical interactions between people is that the interacting people are subjected to power relations, as Dutilh-Novaes notes (2021). She also notes that the work of more senior mathematicians typically attracts more attention, and thus it benefits from more adversarial collaboration. Consequently, their work typically becomes more ‘certified’ and less doubted. In fact, it is not uncommon for mathematicians to believe in theorems without checking their proofs themselves (Geist et al. 2010), and thus testimony can play a central role in mathematical knowledge. At this point it is important to highlight a parallelism between certification and our take on petrification in Sect. 2. Both certification and petrification are processes that increase credibility and decrease doubtability in mathematics. The relevant difference is that the source of certification is *regularities* at the level of dialogical interactions (e.g., a proof tends to resist challenges; this depends on decisions on what should be proved, what or whose work should be addressed, what or whose work should be more or less scrutinized…), while the source of petrification is inner-mathematical *regularities* (e.g. the results we tend to obtain, for instance, at a basic level, by counting, or more generally sustained experiences with mathematical pieces that prove to be useful). Later on, however, we argue that these processes involve a loop that renders this difference merely pragmatic rather than essential.

The phenomenon of certification, arising from dialogical interactions, is also influenced by the characteristics of the proposer. For instance, as noted before, the proposals from senior researchers (acting as proponents) achieve higher certification (and hence less doubtability) among other things because they benefit more from adversarial collaboration. However, seniority is not the only personal characteristic of the proposer that matters in the economy of credibility. Regularities about the reputation of the mathematician due to previous achievements also affect how the community perceives and assesses the mathematician’s work and their expertise (Hanna 1983; Reid 2002; Weber 2008). Their work being interesting, original and/or rigorous may be a regularity itself. As such, certain social identifiers are not just proxies for fundamental phenomena like adversarial collaboration, but have their own net effects in the economy of credibility. Not only do they play a role in deciding what work is worth engaging with or scrutinizing, but they may modulate adversarial collaboration itself: in a given test, they may make a difference and take away from the normativity of one of the propositions of the work under scrutiny, or may modulate how many checks are necessary to achieve a given level of trust, how tolerant one is about presentation issues, etc.^17^

For instance, regarding Mochizuki’s polemic proof of the ABC conjecture (for which there is no consensus insofar as its correctness is still open to debate many after), Rittberg (2021) makes the case that there were presentation issues in Mochizuki’s work, and yet the community tried hard to understand it because of a prior regularity:

He earned the respect of the international mathematical community and is known as a careful and deep thinker. (...) Mochizuki’s prior work commands significant respect and this is at least part of the reason why mathematicians are willing to invest so much effort in understanding his proof. (Rittberg 2021, 5586-5588)

A stronger effect has been reported by Jackson (2006) in her discussion about Perelman’s proof of the Poincaré Conjecture. We find many parallelisms with Mochizuki’s case in this discussion. First, there are presentation issues: a mathematician remarks that Perelman’s articles ‘are not written in such a way that one can just sit down and quickly decide whether his arguments are complete’ (Jackson 2006, p. 898). Second, Jackson comments that Perelman still received considerable attention, in part due to his previous achievements:

Nevertheless, his efforts were from the outset taken quite seriously. One reason is that Perelman is a well-regarded mathematician who had already made distinguished contributions to geometry analysis. (Jackson 2006, p. 898).

Third, there is one aspect that indicates a stronger effect of Perelman’s expertise on the assessment of his work (compared to Mochizuki’s case): some mathematicians believe that Perelman’s proof is correct despite not knowing its details. One mathematician claims that the proof ‘must be right’, in part, because ‘Perelman is a well-known expert on Ricci curvature, and his previous papers have been reliable and have not been found to contain mistakes’ (Jackson 2006, p. 899). Another mathematician reports that he has ‘no doubts that Perelman can also prove the Geometrization Conjecture, but (he) has not personally gone through that proof in detail’ (Jackson 2006, p. 899).

Some empirical evidence suggests that this effect is more prominent the more one is uncertain as to whether one should be persuaded by a given mathematical argument (Inglis and Mejia-Ramos 2009). Perhaps this is the reason why Saitoh’s ‘Division by Zero Calculus’ has been largely ignored by the community, despite Saitoh’s otherwise celebrated career: it leads to evident contradictions (see Ernest 2023 for a discussion on Saitoh’s recent and polemic work). Together with the evidence above, this suggests that, much like the phenomenon of petrification described in Sect. 2, certification also seems to come in degrees and is context-dependent. Its net contribution is to immunize work against criticism, but because it is not the only relevant factor at stake across contexts (others being petrification and the character of challenges presented), it will not always succeed—the more a given work has been certified, the better the chances. Furthermore, certification has a legitimate place in the pragmatics of epistemic practices like mathematics.

**Fig. 1:**
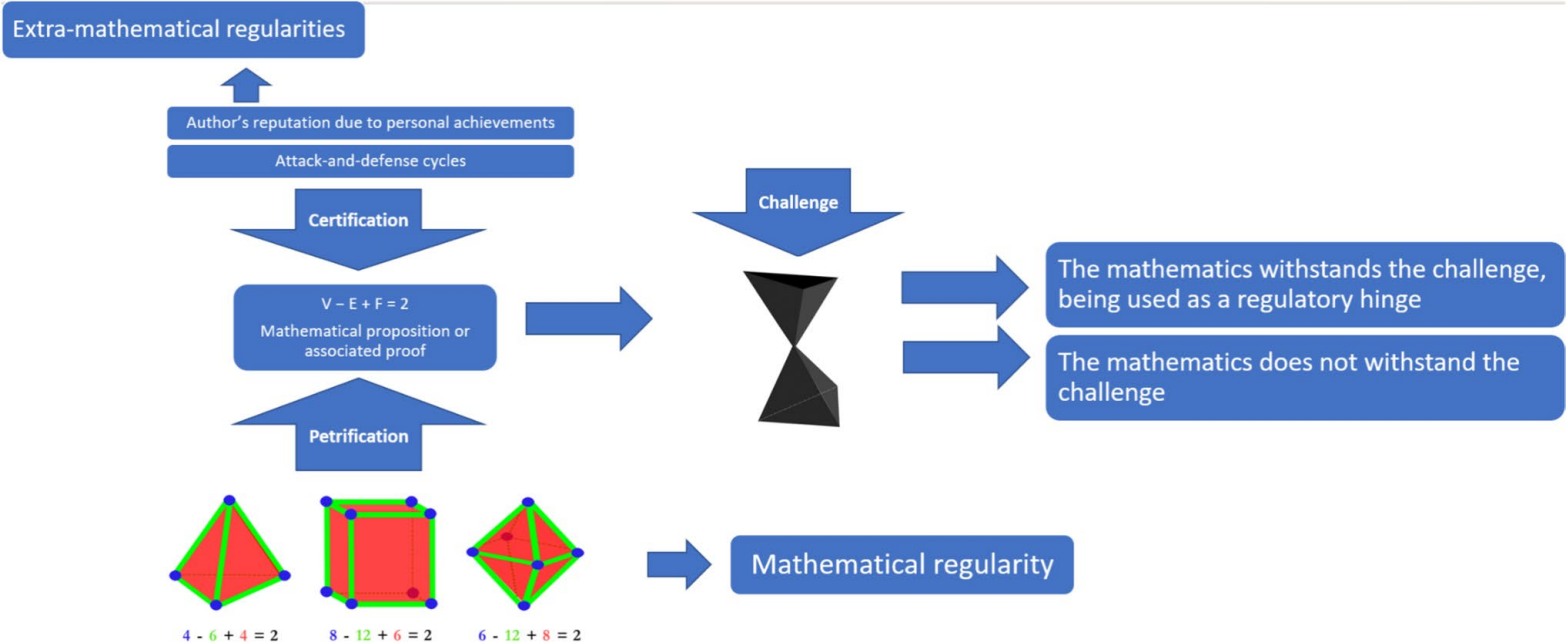
A mathematical regularity petrifies into a mathematical proposition. Extra-mathematical regularities certify the mathematical proposition. Petrification and certification synergize in conferring normative power to the mathematical proposition which may contribute to its withstanding of challenges in certain contexts

Let us recapitulate. Some mathematical *regularities* (like, but not only, the propositions of arithmetic and mathematical models) may petrify and thus become normatively charged. Attack-and-defense cycles polish a work that, in its last form, has *regularly* withstood criticism. The characteristics of a mathematician (e.g., previous performance *regularities*) may certify their output, influencing how it is perceived and assessed. Being grounded in regularities like petrification, certification per se, as described so far, is unrelated to epistemic injustices. Petrification and certification are based on different types of regularites, but are both key components of mathematical practices, and decrease the doubtability of mathematics in degrees and in a context-dependent way. Overall, both certification and petrification fit our general framework of context-dependent mathematical hinges. Before we transition to the next section, where we discuss potentially harmful extra-mathematical hinges, let us cast what we have so far schematically (see Fig. 1).

## Prejudices Hijack Legitimate Certification Processes in the Economy of Credibility

Up until now, we have seen how extra-mathematical regularities can affect the hinge dynamics of mathematical practices in the form of certification of mathematical propositions and proofs. We have also seen that prejudicial-hinges (ungrounded in regularities) and ST-hinges (reliable and unreliable) are relevant in many epistemic practices. In this section, we argue that the pathway by which prejudicial-hinges and unreliable ST-hinges affect mathematical practices, rather than being simple and direct, works by interfering with and exploiting the (otherwise legitimate) process of certification in open-ended mathematical processes like assessing conjectures and proofs. This would explain why, even under the effect of a strong prejudice, we would seldom say that someone who claims that 2 + 2 = 4 (e.g., as a model of apple addition) is wrong: this arithmetical proposition in such a context is probably too petrified for certification to make a difference in an overall economy of credibility.

In the previous section we argued that extra-mathematical factors like social identifiers can affect certification processes in two ways: 1) affecting the chances of engaging with certain work (and thus affecting occurrence of certification processes to some extent) and 2) modulating the effectiveness of certification processes like adversarial collaboration because they affect the attitudes of the epistemic agents involved. We also said that this does not necessarily entail epistemic injustice. However, there is an exception to this, which we call ‘certification hijacking’: some social identifiers may have effects that are not properly grounded on regularities or their scope is abused. Such a hijacking can illegitimately increase or decrease the degree of certification of a mathematical piece: while a prejudicial-hinge on a proposer decreases certification (‘undercertifies’), an unreliable ST-hinge increases certification (‘overcertifies’). Reliable ST-hinges, on the other hand, contribute to certification processes legitimately because they are grounded on reliable empirical generalizations and their scope is not abused.

We must also note that the process of certification can be negatively affected (in the sense of attaining ‘undercertification’) in legitimate ways. One of them, also grounded in a regularity, is the case of a negative track record of a given mathematician. However, it is hard to assess cases of effects of bad reputation in the economy of credibility. It is relatively easy to find cases where people admit their assessments to be influenced by an outstanding track record. However, instances of the opposite are much harder to come by. There are obvious social barriers to openly admitting that one discredits a given mathematical output because its author has been shown to be unreliable in the past. Furthermore, as we saw before, the assessor is not always aware of the effects of reputation in their assessment (this also applies to instances of good reputation). Despite this initial difficulty, there are at least two reasons to argue that a bad reputation may be detrimental in the economy of credibility (in extreme cases leading to a ‘negative’ hinge use of the mathematics, i.e. against its correctness, disregarding empirical information or proof-testing). The first is rational: if a historical regularity about good performance can add to the economy of credibility and sometimes lead to a ‘positive’ hinge (for the correctness of the mathematics), then it is reasonable to expect that a historical regularity about bad performance may take away from the economy of credibility and promote an opposite hinge. Of course, this reason by itself may not be enough. The second reason is that, as we saw in Sect. 3, prejudices do have this effect even in the absence of this kind of bad track record regularity (thus hijacking certification processes). Conversely, a bad track record does not necessarily involve prejudice, but these two reasons render the effects that we propose highly plausible.

There is also the more problematic case of the assessment of students. Students typically lack substantial track records in mathematical research. As such, one may argue that there is no regularity affecting certification processes. For the most part, students lack career reputations: there is no substantially long regularity of a given student’s performance, unless we take into account how the student has performed relative to other students. One may also appeal to the regularity that students typically perform worse than established research mathematicians in research mathematics (i.e., an epistemically reliable stereotype). Be it as it may, it is known that the mathematical arguments of students are not assessed like those of established mathematicians (Inglis and Mejia-Ramos 2009). This highlights a second difficulty: not only is it hard to come by documented cases of certification hijacking, but it is also unclear if certification hijacking, in the case of students, happens because of negative prejudices or because of a (perhaps more reasonable and reliable) lack of the positive perception of more established mathematicians. These two scenarios would affect certification processes differently and, like in the case above, it is hard to discern which one of the two (if any) is responsible for effects in certification processes.

Despite these difficulties, we can establish a demarcation: a bad track record or a lack thereof do not necessarily involve prejudice. As we saw in Sect. 3, prejudice-hinges about the social identity of the proposer may lead to a credibility deficit. Such a credibility deficit may prevent engagement with the proponent’s knowledge contribution altogether, despite a lack of evidential grounds to do so. Prejudice-hinges are different from the kind of bad reputation described above in that there is no evidence of past regular bad performance by the proposer, but instead the critic attributes unreliability to the social identity of the proposer.^18^ Yet, prejudice-hinges and bad reputation are similar in that, at least in some epistemic practices like mathematics, they lack a deterministic effect. This is because, as we have argued, there are more intertwined factors in the economy of credibility.

**Fig. 2:**
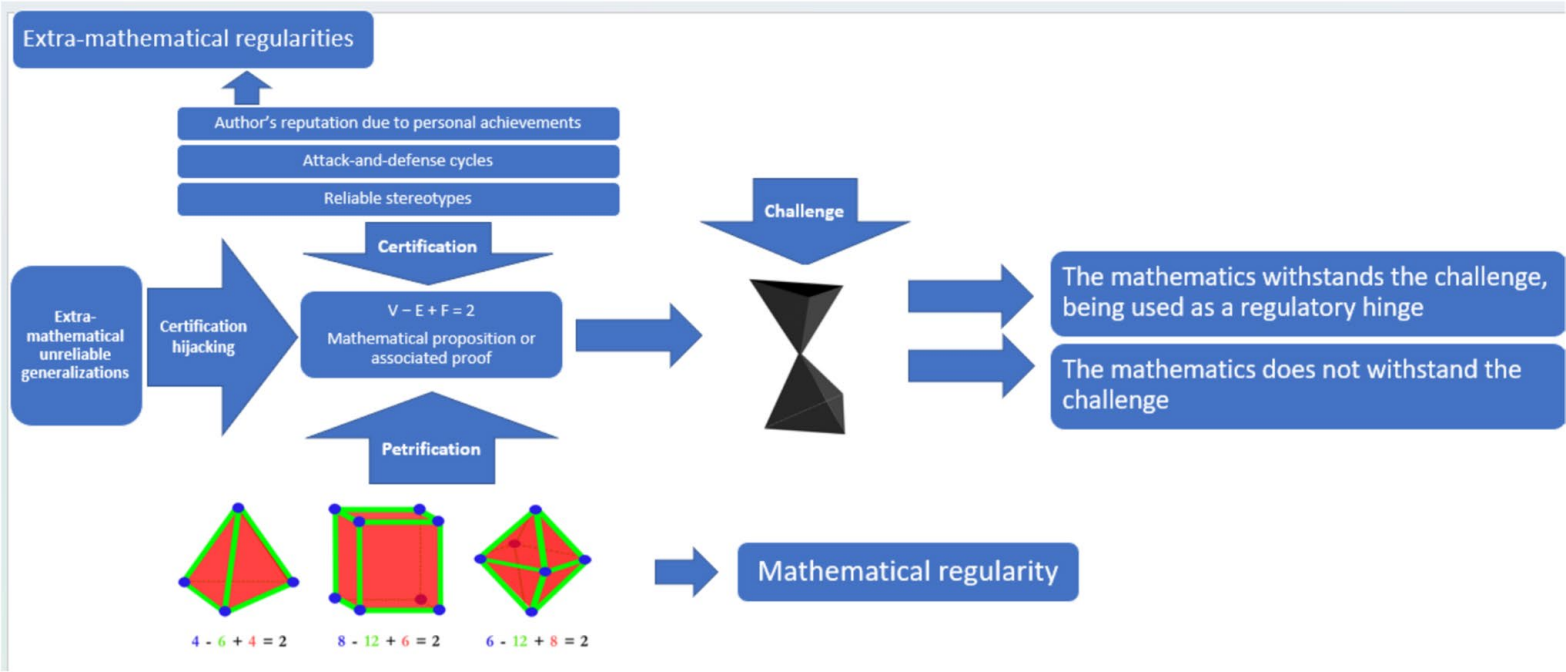
Prejudice-hinges and unreliable ST-hinges (in the form of unreliable generalizations) may compromise the legitimate and necessary contributions of certification (for instance, the benefits of adversarial collaboration, and to some extent of reliable ST-hinges) and petrification of inner-mathematical regularities. Although not depicted in the figure, certification and certification hijacking processes also happen at the level of challenges to the mathematical piece

For instance, if the net effect of petrification in the overall economy is very high, then the net effect of extra-mathematical factors becomes relatively less relevant (in other words, they become ‘diluted’). However, this also renders the effect of extra-mathematical factors harder to acknowledge. There are more difficulties in the identification of the effects of prejudice-hinges, even when certification processes are more relevant (i.e., when petrification is not almost a fully deciding factor). Even if certification processes play a role in what to do in a given case, one can just declare the outcome as the natural outcome of intra-mathematical processes. We have made the case that mathematical practices are open-ended to some extent, which means that there is no intra-mathematical neutral arbiter which can be used as a reference to assess deviations from purely intra-mathematical standards. This, together with the fact that the net effect of extra-mathematical criteria is hard to determine in specific cases, one cannot readily blame prejudices for a given mathematical outcome.^19^ We believe that this mechanism explains why it is hard for recent philosophical work to ascertain whether there has in fact been epistemic injustice in assessing mathematical work in specific cases.^20^ Furthermore, thorough case studies in this direction are not as abundant as in other philosophical issues,^21^ which prevents such indeterminacy from being better characterized. We hope that our schematic presentation based on the integrated hinge epistemology of mathematics (consisting in the synergy of both inner-mathematical and extra-mathematical normative principles) is a first rational step in this direction and motivates further detailed case studies. We present the updated mechanism schematically next (Fig. 2).

If this is correct, mathematics is not just petrified inner-mathematical regularities plus some sort of social ‘gloss’, but a conglomerate with interrelated intra- and extra-mathematical factors. In turn, what we call the inner-mathematical processes that lead to petrification may also be the result of previous economies of credibility where extra-mathematical factors were involved. As such, the division between intra- and extra-mathematical factors is not meant to be strict, but rather pragmatic in the context of this work. Furthermore, note that, just like hinges can work against each other (Boncompagni 2024; cf. Medina 2013 on epistemic friction), they can work with each other, synergizing in conferring normative power to a mathematical piece against more challenges in more diverse contexts. Thus, the psychosocial processes involved in certification processes are not mere external complements to mathematical practices, but a constitutive part of mathematical practices. Therefore, mathematical practices themselves are not ethically neutral. As such, this hinge epistemological framework complements previous literature in the relationship between social and ethical issues and mathematical practices (e.g., Rittberg et al. 2020; Tanswell and Rittberg 2020; Tanswell and Kidd 2021; Hunsicker and Rittberg 2022; Müller et al. 2022; Pérez-Escobar and Sarikaya 2022; Braun 2023; Ernest 2023; Rittberg 2023a; Wagner 2023). For instance, Rittberg et al. (2020) discuss that there are participation barriers in mathematics. While some of these barriers are legitimate and necessary (Rittberg 2023b), others are opaque and can thus be biased and exploited, and lead to epistemic injustice. The open-endedness of the hinge configurations in mathematical practices that we discuss is tacit and often suffers from opacity, and thus, it is a potentially exploitable feature leading to unfair participation barriers.

Last, the reader may believe that the framework here depicted is relevant to areas of knowledge other than mathematics with some adaptations (for instance, substituting the process of mathematical petrification with other principles involving regularities), and this is certainly the case. However, this framework is especially relevant to mathematics. This is due to some distinctive features of mathematics that facilitate and/or mask the hijacking of certification processes. First, mathematics distinctively features hinges with very strong normativity. In fact, *On Certainty* engages very often with mathematics for this reason. Second, mathematics, especially pure mathematics, enjoys a social perception of epistemic neutrality to a higher degree than other disciplines (see, e.g., Wagner 2023). Third, as Dutilh-Novaes (2021, p. 210) discusses, mathematical peer review is different from that of other areas: there is typically a single reviewer and the review is typically not blinded (i.e., the reviewer knows the identity of the authors but not the other way around). Furthermore, articles are often prepublished in arXiv, and thus the identity of the author is disclosed in the first place.^22^ In fact, in some mathematical areas there are only a few active research mathematicians. Further note that recent scholarship shows that peer review in top mathematical journals is typically preceded by ‘quick opinions’: senior mathematicians provide the editors with a quick verdict based on interestingness rather than soundness (Greiffenhagen 2024). These particularities often limit the effectiveness of simple solutions against biases. For instance, the third particularity renders anonymization almost ineffective for most contexts of research mathematics. Some contexts, like conference talks and interviews, can only be anonymized in some stages. For instance, anonymization may work when assessing abstracts or the textual part of grant proposals, and even here blinding may be challenging given that many fields of research mathematics involve a limited number of active mathematicians.

## Conclusion

Concluding, in this work we have provided a rational mechanism by which mathematical hinges are formed by the influence of both intra- and extra-mathematical factors. We have uptaken a contextual characterization of hinges (i.e., a mathematical piece is used as a hinge in some contexts but not others) and have further built on it: different factors confer or remove normative power from mathematical pieces in a broad economy of credibility. By doing so, we have unified two hinge epistemologies: that of mathematics and that of testimony. We have argued that, in what we call ‘certification processes’, epistemically legitimate and illegitimate regularities (in the form of the track record of individuals, stereotypes and prejudices, among others) unfortunately meet in an overall economy of credibility, and thereby the illegitimate ones may hijack otherwise legitimate and necessary certification processes in mathematical practice.

The distinctive characteristics of peer review in mathematics and the open-endedness of the tacit, contextual and opaque hinge configurations in mathematical practices diminish the effectiveness of simple measures to ameliorate the hijacking of certification processes. Not only can this manifest into opaque participation barriers and epistemic injustice, but it also affects the hinge dynamics of mathematics and thus mathematics itself. Thus, understanding the phenomena here characterized, especially how hinges behave in mathematical practices via the convergence of intra- and extra-mathematical processes, would help develop more effective, appropriately informed measures to ameliorate both epistemic injustice in social contexts and epistemic harm within mathematics. Future work will engage with this task.

## Data Availability

Not applicable.

## Footnotes

1 Note that hinges are *necessary*, but not *sufficient*, conditions for the success of cognitive projects. Although hinges enable the proper functioning of the epistemic operations at stake, there are many other elements that could factor into the success of a cognitive project. For example, the epistemic agent must properly carry out the appropriate epistemic operations for their cognitive project to be successful. If the epistemic agent makes a mistake, then their project will fail (despite there being the necessary hinges to ensure the proper functioning of the required epistemic operations).

2 Hinge epistemologists remain at odds over whether hinges are exempt from epistemic appraisal or not. Moyal-Sharrock (2004), Coliva (2015) and Pritchard (2016) hold that hinges are beyond doubt and justification, while Wright (2014) and Hazlett (2014) hold that hinges can be potentially justified, albeit non-paradigmatically.

3 Note, however, that this axiom may not be a hinge in all contexts, for instance, when working with certain conceptions of infinity (cf. note 8). A notable case is set theory. This further shows that axioms can forfeit their status as mathematical hinges in some contexts while retaining it in others.

4 For more on the notion of petrification in mathematics, we refer the reader to Fogelin (1995), Steiner (2000; 2009), Bangu (2012), Kusch (2016) and Pérez-Escobar (2022; 2023).

5 In fact, in Lakatos’ account, Euler did not feel the need to prove his conjecture for polyhedra: by observing many instances of polyhedra, he concluded that the conjecture held true. Moreover, Euler established new and groundbreaking (in the sense of departing from previous grounds of mathematics) mathematical practices in his time, like dividing by 0 to obtain infinity.

6 There are multiple forms of epistemic injustice that will not be considered here. See Kidd et al. (2017) for an overview.

7 It is unclear whether Boncompagni’s comparison is drawn between hinges and (i) all identity-prejudices or (ii) only those that implicitly regulate testimonial justification in epistemic exchanges. For the purposes of this paper, we restrict the hinge-account solely to the latter (cf. footnote 8).

8 As a reviewer rightly notes, prejudices (and implicit biases more generally) need not always serve the function of hinges, i.e., fundamental presuppositions that are exempt of doubt and regulate our behavior in a domain. At times, prejudices are discussed explicitly, put in doubt and even rejected by individuals who then assume them implicitly in their behavior elsewhere. This duality need not pose a threat to our work here. Many hinge epistemologists (see e.g., Wright 2004; Moyal-Sharrock 2004; Kusch 2016; Fairhurst et al. 2024) take hinges to be a context-sensitive notion. Namely, the status of hinges is specific to either a particular domain of inquiry or cognitive project (see Johnson 2020 for a detailed explanation). For example, while ‘The world did not come into existence five minutes ago’ is a hinge in the domain of history or physics, it is just a mere truth-apt proposition open to doubt in other domains, such as philosophical skepticism. Our contention is that this holds too for prejudices. Moreover, Boncompagni (2019; 2024) has sought to exploit this context-sensitivity to explain how one may combat hinge-prejudices.

9 Coliva’s testimonial-hinge describes the *minimal* conditions required to attain testimonial justification. As such, it does not exclude scenarios where further (non-essential) conditions are met (see Sect. 3.2).

10 As a reviewer rightly notes, one may suspect that this characterization of hinges is at odds with our earlier description. In this example we show that Dani has a hinge that Alex does not share, seemingly misaligning with our earlier characterization of hinges as commitments. To avoid this confusion a clarification is in order. Although hinges remain essentially communal, this does not entail that *all* hinges *must* be universally held. There is a broad literature showing how some hinges are only locally shared by specific communities, such as that discussing the case of deep disagreements where conflicting hinges collide in a disagreement (see Ranalli 2020 for an overview). The size of the community need not extend to all human beings, it can be limited to a subset of them. For example, only the community of scientists will hold certain rules of evidential significance (e.g., certain complex methods of evidence acquisition) that are not shared among those who are not within this scientific community. So, while Dani may pertain to a community that holds a prejudicial-hinge about women of color, Alex may not be part of this community and, by extension, they will not hold Dani’s prejudicial-hinge.

11 The precedence and preemption of prejudicial-hinges are not chronological (e.g., prejudicial beliefs acquired at childhood), but normative. ‘It normatively precludes the hearer from considering the speaker’s words as evidence and the corresponding belief as justified […] by substituting the ordinary testimonial hinge that would otherwise regulate credibility assessment and justification’ (Boncompagni 2024, pp. 295–296).

12 Moreover, identity-prejudices can exhibit other diverse features. For instance, they may be (i) reliable or unreliable, (ii) beliefs or other types of cognitive commitments (e.g., implicit presuppositions), and (iii) strong (e.g., all X are Y), weak (e.g., some X are Y) or something in between (e.g., many X are Y).

13 Our proposal solely concerns positive identity-stereotypes fulfilling a specific kind of cognitive commitment: implicit presuppositions that are not susceptible to doubt or skepticism (cf. Kahneman and Tversky 1973; Tversky and Kahneman 1974; Kay et al. 2013; Boncompagni 2024, 295; Coliva 2015; 64). We do not intend to provide a theoretical framework covering all positive identity-stereotypes, such as those functioning as ordinary beliefs (cf. footnote 10).

14 Note that our proposal does not intend to capture all testimonial justifications, only those involving positive identity-stereotypes.

15 If these factors are part of an overall economy they can, for instance, make a difference ‘at the margins’, especially in continuous phenomena like normativity, trust and doubtability. Marginal effects analyses are common in economics but not in domains involving social identifiers or epistemology, let alone a combination of both. Basically, marginal effects refer to the effect on certain outcomes that small changes (‘unit changes’) in one variable, while all other variables are held constant, have. When not explicitly looking for them, marginal effects are easily overlooked, especially when continuous outcomes (like doubtability) are assessed in discrete terms (sometimes even in binary or ‘all or nothing’ terms, like a final verdict on a grant or the publishability of a paper) because they may not make a difference in many cases. This may erroneously lead to overlooking the net effect of these factors on continuous phenomena, only big changes in the variable under scrutiny being visible.

16 Note that this is a useful simplification for the context of this work, since not all hinges are of the same kind, play equal epistemic roles, nor have the same normative power (Fairhurst et al. 2024). Here, we are only concerned with whether a mathematical piece is used as a hinge or not.

17 When these social identifiers are not reliable, this ‘hijacks’ certification processes (see Sect. 5).

18 In fact, in line with the observation in Sect. 4 that prejudices stem from unreliable empirical generalizations, prejudices can also lead to *dysfunctional regularities*, in the sense that a regularity may unfairly regulate a situation outside of its proper context. Paradoxically, this may circularly lead to a regularity, which, even if it becomes epistemically reliable at some point, is dysfunctional from an ethical perspective. For instance, women may have more intense anxiety responses than males doing mathematics (this literature is extensive and lacks definite conclusions, but for the sake of this hypothetical example, see Devine et al. 2012), but this may unfairly regulate our perception of the activity of women that perform highly (and, circularly, induce a certain propensity to anxiety responses that may partially account for the initial regularity instead of being fully accountable by other facts). Similarly, media depictions or salient individual experiences badly generalized may catalyze dysfunctional regularities.

19 An added difficulty is that prejudices are very often unconscious processes (See Sect. 3.1 and foonote 8), and even when people are conscious about their prejudices, it is unlikely that they will express them publicly or cite them as a reason grounding their epistemic practices.

20 A prominent example is that of Olivia Caramello, discussed at length in Rittberg et al. (2020). The authors refrain from affirming that there was epistemic injustice, claiming that ‘we refrain from doing so because there may be relevant information for such a judgement which is inaccessible to us’ (p. 3881).

21 However, for a sampler, the reader is referred to Hunsicker and Rittberg (2022) and Rittberg (2023b).

22 More specificities are that reviewers of mathematical articles are tacitly presupposed to adhere to the so-called ‘Littlewood’s precepts’, which include not just correctness but also more subjective criteria like novelty and interestingness. The evaluation of these criteria is often influenced by legitimate heuristics like the reputation of the mathematician in question (Geist et al. 2010). These criteria also come with their distinctive manifestations and exploits in mathematics (e.g., Hunsicker and Rittberg 2022).
